# A case of pericarditis in a middle‐aged woman with COVID‐19

**DOI:** 10.1002/ccr3.6769

**Published:** 2022-12-20

**Authors:** Hisako Kushima, Sayaka Shimizu, Yohei Koide, Akira Kawamura, Hiroshi Ishii

**Affiliations:** ^1^ Department of Respiratory Medicine Fukuoka University Chikushi Hospital Chikushino Japan; ^2^ Department of Infection Control and Prevention Fukuoka University Chikushi Hospital Chikushino Japan; ^3^ Department of Cardiovascular Diseases Fukuoka University Chikushi Hospital Chikushino Japan

**Keywords:** case report, coronavirus disease 2019, pericardial effusion, pericarditis, remdesivir

## Abstract

The frequency of pericarditis as a complication in COVID‐19 patients without underlying disease is not well known. We report a case of COVID‐19 presenting with pericarditis without myocarditis or severe respiratory symptoms in a middle‐aged woman, who had neither underlying disease nor previous diagnosis of COVID‐19.

## Graphical abstract

We need to be aware that COVID‐19 can present with pericarditis without myocarditis or severe respiratory symptoms even in the absence of underlying disease.

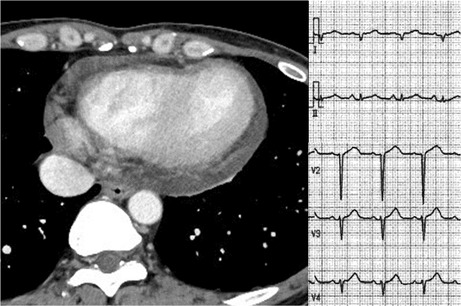

## 1 INTRODUCTION

Coronavirus disease 2019 (COVID‐19) caused by the Severe Acute Respiratory Syndrome Coronavirus 2 (SARS‐CoV‐2) is a highly infectious disease. In COVID‐19, histopathological changes have been reported in various organs. Although several studies have reported the detection of SARS‐CoV‐2 in nonrespiratory organs, to date, it is not clear whether SARS‐CoV‐2 can directly infect and cause histopathological changes in organs other than the respiratory tract. In addition, pulmonary, cardiac, arthritic, cutaneous, and other organ damage may also develop as complications after COVID‐19.^1^ Ahmed et al.^2^ reported that endothelial dysfunction, platelet activation, hyperviscosity, and blood flow abnormalities due to hypoxia, immune reactions, and hypercoagulability can lead to thrombogenesis in COVID‐19. However, it is still unknown whether nonrespiratory lesions are due to direct tissue damage from SARS‐CoV‐2 infection or to the host's response to infection.

The clinical spectrum of COVID‐19 ranges from asymptomatic infection to life‐threatening multi‐organ failure. The cardiovascular system is increasingly recognized as an important target of SARS‐CoV‐2 infection, leading to acute coronary syndrome with or without obstructive coronary artery disease, arrhythmias, venous thromboembolism, myocarditis, and pericarditis.^3^ The incidences of myocarditis, pericarditis, and cardiomyopathy have been reported to range from 0.098% to 3%,^4^, ^5^ and a meta‐analysis of 2676 hospitalized patients with confirmed COVID‐19 showed a pooled prevalence of pericardial effusion of 3% on chest computed tomography.^6^ However, the frequency of pericarditis alone as a complication in COVID‐19 patients without underlying disease or previous SARS‐CoV2 infection is not well known.^7^, ^8^

## 2 CASE REPORT

A 41‐year‐old woman without any underlying diseases was rushed to our hospital due to loss of consciousness in a sitting position. She had never been vaccinated against SARS‐CoV‐2 and had no family history of cardiac diseases. A family member living with the patient developed COVID‐19, and she developed fever 6 days later and was diagnosed with COVID‐19. She had a persistent fever and also developed sore throat and chest pain with an uneasy sensation on the fifth day after the onset.

**FIGURE 1:**
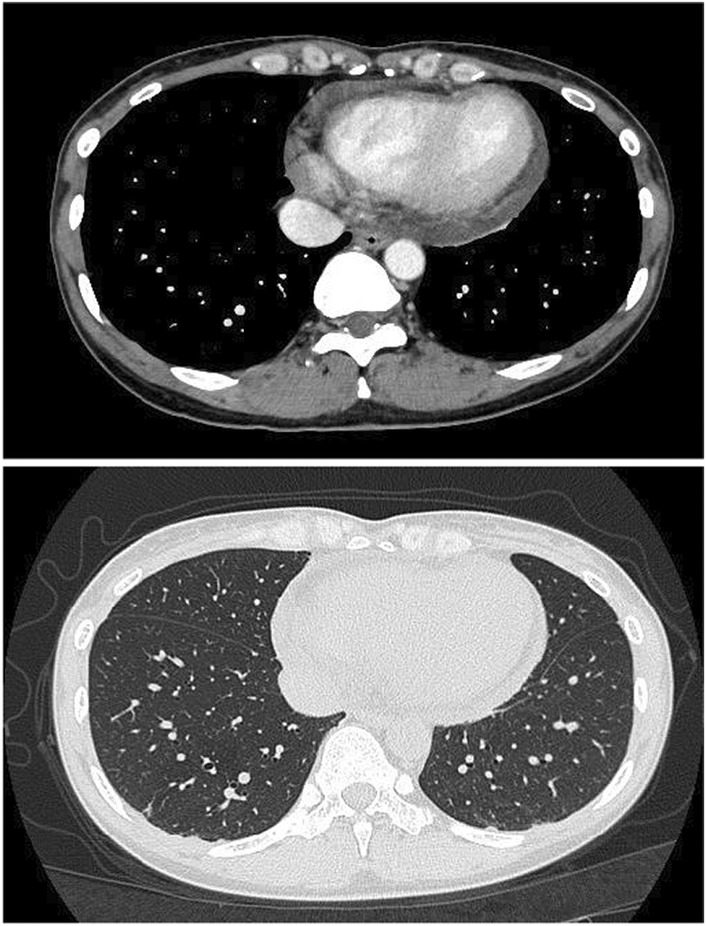
Chest computed tomography on admission showing significant pericardial effusion without infiltrates in both lungs.

On examination, her heart rate and blood pressure were 93 beats/min and 86/64 mmHg, respectively, her respiratory rate was 21/min, her body temperature was 36.0°C, and the O_2_ saturation at room air was 97%. By the time she arrived at the hospital, she had already regained consciousness but had persistent mild chest pain. The patient did not present with a skin rash suspicious of other viral diseases such as herpes virus. Chest computed tomography revealed significant pericardial effusion without infiltrates in both lungs (Figure 1). Transthoracic echocardiography also showed moderate‐volume pericardial effusion around the posterior left ventricular wall and apex, and it revealed more than 60% of ejection fraction without any valve abnormality. Electrocardiography (ECG) showed PR depression and ST elevation in the lead І, II, and V2–V6 with lower amplitude in the limb lead. Repeated ECG after 1 h revealed the same finding. At our institution, we were unable to perform an evaluation based on cardiac magnetic resonance imaging of COVID‐19 patients.

The serum levels of troponin T, creatine kinase MB, lactate dehydrogenase, thyroid stimulating hormone, free thyroxine, ferritin, and interleukin‐6 were normal, and the pro‐brain natriuretic peptide was 306 pg/mL (normal <54). Other blood investigations revealed a hemoglobin level of 14.4 g/dl, total leucocyte count of 8800/μl, platelet count of 16.3/μl, and C‐reactive protein of 0.18 mg/dl (normal <0.14). The coagulation profile was normal. Autoimmune‐associated serum markers, such as antinuclear antibody and rheumatoid factor, were also negative. Interferon‐gamma release assay and hepatitis B surface antigen were negative. There was no background suggestive of human immunodeficiency virus infection.

Furthermore, we investigated the presence of SARS‐CoV‐2 in the serum taken on the day of admission. After the blood was centrifuged, the viral ribonucleic acid (RNA) was extracted using the SMITEST EX‐R&D kit (MBL) and quantified by quantitative reverse transcription PCR (RT‐qPCR) (Thermo Fisher Scientific). Primers and probes targeting the nucleocapsid gene, namely 2019‐n‐CoV‐N‐F2 (position nt29, 125–nt29, 144), NIID 2019‐n‐CoV‐N‐R2 (position nt29, 299–nt29, 280) and NIID 2019‐n‐CoV‐N‐P2 (position nt29, 222–nt29, 241) were used. These were developed by the Japan National Institute of Infectious Diseases. The full method of RNA quantification has already been described.^7^ As a result, her serum was positive for SARS‐CoV‐2. Other possible viral infections such as coxsackie virus infection that can cause pericarditis were ruled out by the results of several serological tests. Thus, her pericardial effusion was considered to be due to COVID‐19 infection.

**FIGURE 2:**
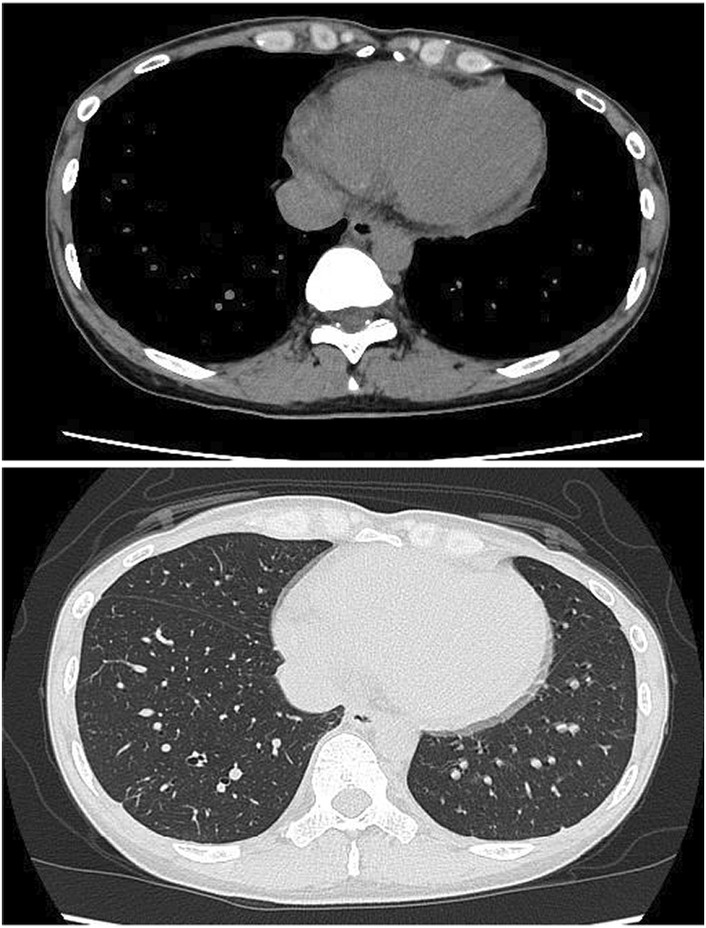
Chest computed tomography after treatment showing decreased pericardial effusion.

She was started on oral high‐dose aspirin (1500 mg daily) and intravenous remdesivir immediately upon admission (200 mg loading dose on day 1, followed by 100 mg daily for 4 additional days). Although there was no worsening of oxygenation or development of pneumonia, the patient was given remdesivir because of complications of pericarditis and concern that her general condition would deteriorate. Her chest pain rapidly decreased, and no subsequent loss of consciousness occurred. Her body temperature never exceeded 37.0°C since admission. The pericardial effusion had disappeared on CT (Figure 2), and the serum SARS‐CoV‐2 findings turned negative on the 10th day. Although the administration of dexamethasone or colchicine was considered if the patient's symptoms worsened, these medicines were not needed. She was able to be discharged from the hospital without any complications on the 11th day.

## 3 DISCUSSION

Thus far, no diagnostic or treatment guidelines for COVID‐19‐associated pericarditis have been developed, due to the limited amount of data. Most reported cases have been associated with myocardial involvement with elevated levels of serum troponin and either the presence or absence of pericardial effusion.^9^ We reported a case of COVID‐19‐associated pericarditis with pericardial effusion and viremia but without elevated troponin levels or myocarditis.

The prevalence of detectable inflammatory heart disease in professional athletes with prior COVID‐19 infection in the United States was reported to be 0.6% (5/789), and 2 of those 5 patients had pericarditis. All patients were accompanied by mild respiratory symptoms, but none of them were clinically assessed as having severe COVID‐19.^5^ By contrast, in Japan, myocarditis, pericarditis, and cardiomyopathy were reported to occur at rates ranging from 0.098% to 3%, and these entities were significantly associated with congestive heart failure, mild diabetes mellitus, metastatic tumor, and collagen disease.^4^ However, the frequency of pericarditis alone is estimated to be much lower. In a review of pericarditis in patients with COVID‐19 published in 2022, abnormal lung parenchyma on chest imaging (X‐ray or computed tomography) was reported in 60% of cases.^10^ Therefore, the present case was considered rare and unusual, as the patient had neither respiratory symptoms, abnormal chest shadows nor underlying diseases.

The pathogenesis of acute pericarditis and myopericarditis in COVID‐19 patients is still poorly understood. Dysregulation of the immune system is key in the pathogenesis of SARS‐CoV‐2 infection, leading to an overproduction of pro‐inflammatory cytokines in some patients and resulting in what has been termed cytokine storm.^11^ This increased inflammatory response may play a role in the different cardiovascular presentations associated with COVID‐19, including pericarditis and myopericarditis.^11^ A definitive diagnosis is usually lacking, as it requires the detection of the viral genome in the pericardial fluid or tissue through invasive methods, which are generally not used in routine clinical practice. Furthermore, viral serological tests have not proven to be clinically useful, as they only suggest a recent viral infection with little impact on treatment and have been shown to have a poor correlation with the detection of viral genomes in pericardial fluid.^12^ However, Ouoba et al.^13^ described that viremia caused by SARS‐CoV‐2 was observed in all COVID‐19 patients at admission and that viral copies gradually dropped to undetectable levels in patients with mild symptoms but fluctuated and remained persistent in moderate patients. The present findings suggest that the severity of infection and degree of viremia are related, as the disappearance of viremia reflected the clinical course of our patient's recovery.

COVID‐19‐associated pericarditis or pericarditis as one phenotype of COVID‐19 is relatively rare. However, it may be present in cases that would otherwise have mild COVID‐19 without pneumonia or hypoxemia. Chest discomfort or mild chest pain followed by respiratory symptoms, such as sore throat and cough, might be considered an initial manifestation of viral pericarditis, including in cases of COVID‐19. Physical findings, such as hypotension and tachycardia, pericardial effusion on chest CT, and abnormalities on ECGs should be noted, and echocardiography and serological virology should be performed as needed.

A previous review showed that there have been a few cases in which remdesivir was administered to patients with COVID‐19‐associated pericarditis.^10^ Although whether or not remdesivir directly improved pericarditis caused by SARS‐CoV‐2 is unclear, at least in the present case, the combination of aspirin and remdesivir resulted in the disappearance of viremia and pericardial effusion.

We encountered a case of COVID‐19 complicated with pericarditis in a middle‐aged woman without underlying disease. Clinicians should be aware that COVID‐19 may present with pericarditis without myocarditis or severe respiratory symptoms and once again keep in mind that any organ of the body can be affected. In the future, more research should be done on how to make a definitive diagnosis of SARS‐CoV‐2 pericarditis and how to treat it.

## AUTHOR CONTRIBUTIONS

**Hisako Kushima:** Conceptualization; investigation; methodology; supervision; writing – original draft; writing – review and editing. **Sayaka Shimizu:** Supervision; writing – review and editing. **Yohei Koide:** Investigation; writing – review and editing. **Akira Kawamura:** Supervision; writing – review and editing. **Hiroshi Ishii:** Supervision; writing – review and editing.

## CONFLICT OF INTEREST

On behalf of all authors, the corresponding author states that there is no conflict of interest.

## DATA AVAILABILITY STATAEMENT

Data sharing is not applicable to this article as no datasets were generated or analyzed during the current study.

## ETHICAL APPROVAL

Ethics approval was not obtained due to clinical standard treatment.

## CONSENT

Written informed consent was obtained from the patient for publication of this case and any accompanying images.
